# Feasibility of Therapist-Guided Personalized Music Listening for Fibromyalgia: A Pilot Study of Self-Directed Home-Based Intervention

**DOI:** 10.3390/healthcare14152397

**Published:** 2026-08-04

**Authors:** Takiko Takahashi, Masahiro Sugimoto

**Affiliations:** 1 Music Therapy R&D Center, Tokyo 174-0071, Japan; 2 Graduate School of Media and Governance, Keio University, Fujisawa 252-0882, Japan; msugi@sfc.keio.ac.jp; 3 Institute of Medical Science, Tokyo Medical University, Tokyo 160-8402, Japan

**Keywords:** music listening, fibromyalgia, feasibility, self-directed intervention, autobiographical memory, chronic pain

## Abstract

**Highlights:**

**What are the main findings?**

- Therapist-guided, self-directed personalized music listening was feasible in patients with fibromyalgia.
- Responses varied substantially across individuals, with some participants reporting autobiographical memory recall, positive psychological experiences, and increased motivation.

**What are the implications of the main findings?**

- Personalized music listening may represent a feasible low-burden self-care strategy.
- Individualized and therapist-guided approaches may enhance music-based interventions for chronic pain management.

**Abstract:**

**Background/Objectives**: Music-based interventions have been used to alleviate chronic pain and psychological distress; however, their effectiveness varies across individuals, particularly in real-world settings. The COVID-19 pandemic has further highlighted the need for feasible, therapist-guided but self-directed interventions that can be implemented in daily life. This exploratory pilot study primarily evaluated the feasibility of therapist-guided, self-directed personalized music listening in daily life. Exploratory quantitative and qualitative outcomes were also assessed to inform the design of future controlled studies. **Methods**: Seven patients with fibromyalgia participated in a 4-week music-listening period and a 4-week non-intervention period. The intervention was conducted in participants’ daily living environments during the COVID-19 pandemic. A music therapist guided music selection and listening strategies, while participants implemented the intervention independently. Pain scores were recorded daily, and psychological measures (GHQ-12, JFIQ) and salivary β-endorphin levels were assessed at predefined times. **Results**: The therapist-guided, self-directed intervention was feasible in participants’ daily lives. Individual responses varied. Some participants reported psychological experiences, including autobiographical memory recall, increased motivation, and greater engagement in daily activities. No consistent effects on pain intensity or salivary β-endorphin levels were observed. **Conclusions**: This feasibility pilot study suggests that therapist-guided, self-directed personalized music listening is feasible in daily life. Although consistent quantitative improvements were not observed, some participants reported positive psychological experiences, including autobiographical memory recall, increased motivation, and greater engagement in daily activities. These preliminary findings support the feasibility of this approach and warrant larger, adequately controlled studies incorporating objective adherence monitoring and an appropriate washout period.

## 1. Introduction

Music therapy is a non-invasive intervention that utilizes music to improve physical and psychological health, and its clinical benefits have been reported across a wide range of populations [1,2]. Music has been shown to influence emotional regulation, autonomic function, and stress responses, contributing to reductions in anxiety and depression, improvements in sleep quality, and enhanced quality of life [3,4,5,6]. As a result, music-based interventions have been increasingly applied as complementary approaches in diverse clinical settings, including cancer care, postoperative recovery, and geriatric populations [7,8,9].

Among these applications, the effects of music on pain have been relatively well investigated. Previous studies and meta-analyses have demonstrated that music-based interventions can reduce perceived pain in various conditions, including postoperative pain, chronic pain, and cancer-related pain [7,10]. These analgesic effects are thought to involve multiple psychological and neurocognitive mechanisms, such as attentional distraction, emotional modulation, and expectancy effects [11,12]. The gate control theory of pain provides a theoretical framework for these observations, suggesting that cognitive and emotional processes can modulate nociceptive input at the spinal level [13].

However, the analgesic effects of music are not consistently observed, and considerable interindividual variability has been reported [14]. Factors such as music preference and familiarity may influence outcomes, yet relatively few studies have systematically incorporated these elements into intervention design [15]. In addition, although the endogenous opioid system has been proposed as a physiological mechanism underlying music-induced analgesia, direct evidence in humans remains limited, particularly in real-world settings [1,16,17]. These findings highlight the need for more comprehensive evaluations that integrate psychological, physiological, and experiential dimensions.

In addition to potential effects on pain perception, music may influence psychological well-being through emotional engagement, motivation, and the recall of autobiographical memories [18]. Familiar music is often associated with meaningful life experiences and may promote positive affect, self-reflection, and engagement in daily activities [19]. These broader psychological effects may be particularly relevant when evaluating personalized music-based interventions and are consistent with prior research indicating that familiar music can evoke autobiographical memories and promote emotion regulation across a range of clinical populations [19,20,21].

Furthermore, most previous studies have focused on therapist-guided interventions delivered in structured clinical environments. In contrast, less is known about the feasibility and effects of self-directed music-based interventions implemented in daily life, even when supported by professional guidance. This gap has become increasingly relevant in recent years, as the COVID-19 pandemic has restricted access to in-person healthcare and accelerated the need for feasible, home-based, and self-managed interventions [22].

In this context, the primary objective of the present exploratory pilot study was to evaluate the feasibility of therapist-guided, self-directed personalized music listening in patients with fibromyalgia. Exploratory quantitative and qualitative outcomes were also assessed to inform the design of future controlled studies. Fibromyalgia is characterized by chronic widespread pain and psychological distress, making it an appropriate condition in which to evaluate the feasibility of personalized music listening together with exploratory physical and psychological outcomes. Exploratory outcome measures included pain intensity, psychological status, and salivary β-endorphin levels as a physiological indicator. By implementing the intervention in participants’ daily living environments, the study aimed primarily to assess real-world feasibility. Exploratory analyses also examined individual variability and participant-reported experiences associated with personalized music listening. Qualitative data were collected to explore autobiographical memory recall, motivation, and engagement in daily activities.

## 2. Materials and Methods

### 2.1. Study Design

**Figure 1:**
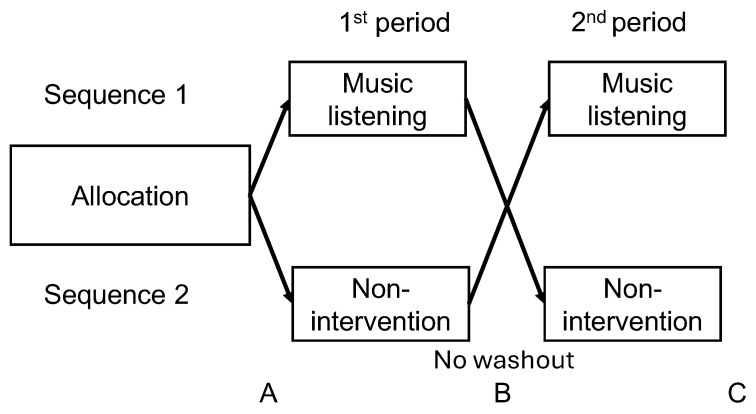
Study design. Participants were assigned to one of two crossover sequences: music listening followed by non-intervention, or the reverse order. No washout period was included between study periods because a pragmatic crossover design was adopted during the COVID-19 pandemic. Assessments were conducted at time points A, B, and C.

This exploratory pilot crossover study was designed to evaluate the feasibility, rather than the efficacy, of therapist-guided, self-directed, personalized music listening (Figure 1). Participants were assigned to one of two sequences: a music listening intervention period followed by a non-intervention period, or vice versa. Each participant completed one 4-week intervention period and one 4-week non-intervention period.

### 2.2. Participants

Seven female patients aged 20–75 years who had been diagnosed with fibromyalgia according to the ACR 2016 criteria [23] were enrolled. Major exclusion criteria included severe medical illness, cognitive impairment, pregnancy or breastfeeding, and changes in analgesic or antidepressant medication within 4 weeks before study entry. Participants continued their regular medications throughout the study period. No major changes in analgesic, antidepressant, or other prescribed medications occurred during participation in the study.

A certified music therapist was involved throughout the study process, including providing explanations during the informed consent procedure, supporting participant enrollment, and coordinating study procedures. The study protocol was approved by the Juntendo University Nerima Hospital Institutional Review Board (N190025; approved on 18 December 2019), and written informed consent was obtained from all participants. The study was conducted in accordance with the Declaration of Helsinki.

### 2.3. Definition of Time-Measuring Points

Three assessment time points were defined as A, B, and C. Each intervention period lasted four weeks. The timing of assessments depended on the intervention sequence, allowing within-subject comparisons between music-listening and non-intervention conditions.

### 2.4. Intervention

During the intervention period, participants engaged in personalized music listening in their daily lives. Participants selected familiar favorite music under the guidance of a certified music therapist.

Familiar favorite music was defined as music that participants particularly liked, had frequently listened to or performed, or associated with personally meaningful memories and life experiences. Such music was selected to facilitate emotional engagement and the recall of autobiographical memories. The same music selections were used throughout the intervention period unless participants specifically requested a change, which did not occur during the study. Listening sessions lasted approximately 30 min and were scheduled twice daily when possible.

A music therapist provided guidance on music selection and listening strategies before the intervention. This included recommendations for engaging in music (e.g., focusing attention, relaxing, or engaging emotionally) and for incorporating listening into daily routines. Participants then implemented the intervention independently in their home environments. The therapist discussed participants’ musical preferences, personal history related to the selected music, and listening situations in daily life before the intervention.

The music therapist monitored adherence through regular follow-up contacts during the study period, confirmed whether participants continued listening to their selected music, and provided support for self-recording of pain and questionnaire completion as needed. However, listening frequency and duration were not systematically quantified because this exploratory pilot study prioritized feasibility in real-world conditions. During the non-intervention period, participants were instructed to maintain their usual daily activities. Concomitant medications were continued without major changes. No washout period was included between the intervention and non-intervention periods because the study was conducted during the COVID-19 pandemic. This pragmatic design was adopted to reduce participant burden and minimize face-to-face contact, although the possibility of carryover effects cannot be excluded.

### 2.5. Pain Score

Pain intensity was assessed using a numerical rating scale (NRS) ranging from 0 (no pain) to 10 (worst imaginable pain) [24]. Participants recorded pain scores once daily at bedtime throughout both study periods. The music therapist provided instructions on recording the data consistently. Period means were calculated for analysis.

### 2.6. Psychological Measures (GHQ-12)

Psychological status was assessed using the 12-item General Health Questionnaire (GHQ-12) [25]. Scores range from 0 to 36, with higher scores indicating greater psychological distress. Assessments were conducted at time points A, B, and C. The music therapist supported participants in completing these assessments as needed.

### 2.7. Disease Impact (JFIQ)

The impact of fibromyalgia was evaluated using the Japanese version of the Fibromyalgia Impact Questionnaire (JFIQ) [26]. Higher scores indicate greater disease burden. Measurements were obtained at time points A, B, and C.

### 2.8. Saliva Collection and β-Endorphin Measurement

Saliva samples were collected between 09:00 and 10:00 under standardized conditions. Participants were instructed to refrain from eating, smoking, and vigorous exercise for at least one hour before collection.

The music therapist provided instructions for saliva collection and supported the procedure as needed. Samples were collected using a Saliva Collection Aid (SCA; Salimetrics LLC, State College, PA, USA) and immediately stored at −80 °C.

Samples were transported on dry ice to a certified external laboratory (Yanaihara Institute Inc., Shizuoka, Japan), where salivary β-endorphin levels were measured using an enzyme-linked immunosorbent assay (ELISA). Results were expressed in pg/mL.

### 2.9. Qualitative Data Collection

Participants were encouraged to describe their experiences with listening to music in their own words during follow-up contacts with the music therapist. The music therapist recorded comments as contemporaneous field notes during follow-up contacts, which were subsequently reviewed and organized into descriptive categories. The music therapist facilitated this process by encouraging participants to reflect on their experiences. Responses were reviewed descriptively and grouped into broad content categories. Because of the exploratory nature of this pilot study, no formal qualitative analytical framework (e.g., thematic analysis) was applied.

### 2.10. Statistical Analysis

Statistical analyses were primarily descriptive because of the exploratory nature of this pilot study and the very small sample size. Individual data were visualized using dot plots and box plots, and no formal hypothesis testing was emphasized in the interpretation of the findings. Missing data resulted from occasional missed self-reports or unavailable saliva samples. No imputation was performed, and all analyses were conducted using only available data. Statistical analyses were performed using Python (ver. 3.12.13) and SciPy (ver. 1.16.3).

## 3. Results

### 3.1. Pain and Quantitative Outcomes

**Figure 2:**
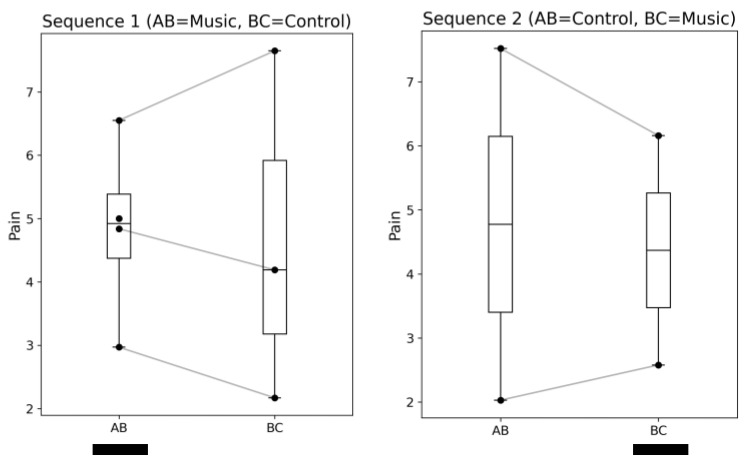
Individual pain scores during the AB and BC periods. Pain scores are presented as individual data points with boxplots for Sequence 1 and Sequence 2. Black circles represent individual participants, and gray lines connect scores from the same participant across the two periods. The boxes represent the interquartile range, the horizontal lines within the boxes indicate the median, and the whiskers extend to the minimum and maximum values. Bold horizontal lines indicate the music listening periods. In Sequence 1, AB corresponds to the music-listening period and BC to the non-intervention period; in Sequence 2, AB corresponds to the non-intervention period and BC to the music-listening period.

Changes in pain scores varied across participants. No consistent reduction in pain was observed during the music listening period compared with the non-intervention period. Individual trajectories demonstrated heterogeneous patterns, with substantial variability across participants and no consistent intervention-related pattern (Table 1 and Figure 2).

**Figure 3:**
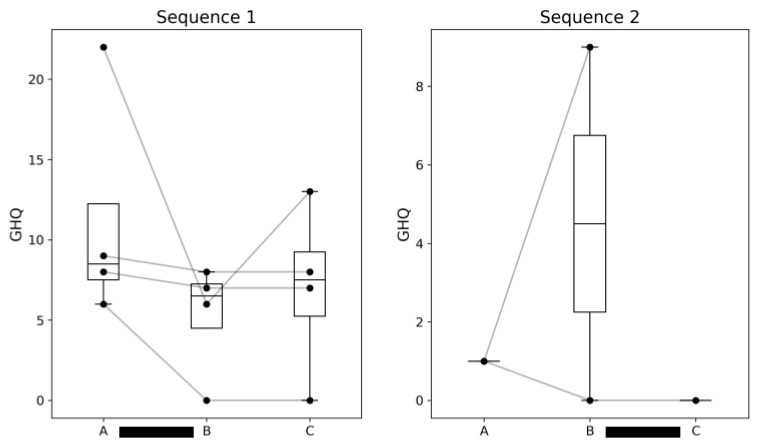
Individual 12-item General Health Questionnaire (GHQ-12) scores at each time point. GHQ-12 scores are presented as individual data points with boxplots at time points A, B, and C for Sequence 1 and Sequence 2. Black circles represent individual participants, and gray lines connect scores from the same participant across time points. The boxes represent the interquartile range, the horizontal lines within the boxes indicate the median, and the whiskers extend to the minimum and maximum values. Bold horizontal lines indicate the music-listening periods (A–B in Sequence 1 and B–C in Sequence 2). Changes varied across participants, with no consistent intervention-related pattern observed. Higher GHQ-12 scores indicate greater psychological distress.

**Figure 4:**
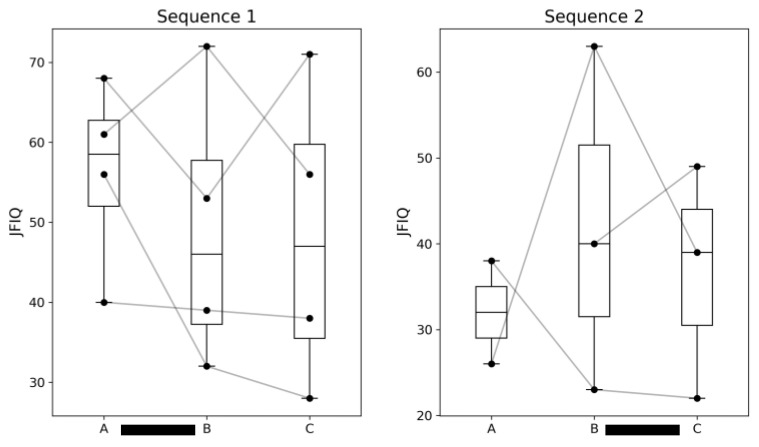
Individual Japanese Fibromyalgia Impact Questionnaire (JFIQ) scores at each time point. JFIQ scores are presented as individual data points with boxplots at time points A, B, and C for Sequence 1 and Sequence 2. Black circles represent individual participants, and gray lines connect scores from the same participant across time points. The boxes represent the interquartile range, the horizontal lines within the boxes indicate the median, and the whiskers extend to the minimum and maximum values. Bold horizontal lines indicate the music-listening periods (A–B in Sequence 1 and B–C in Sequence 2). Scores varied across participants, with no consistent intervention-related pattern observed. Higher JFIQ scores indicate a greater impact of fibromyalgia on daily functioning and well-being.

Changes in GHQ-12 and JFIQ scores varied across participants, with no consistent intervention-related pattern (Figure 3 and Figure 4). Overall, the quantitative findings demonstrated substantial interindividual variability.

**Figure 5:**
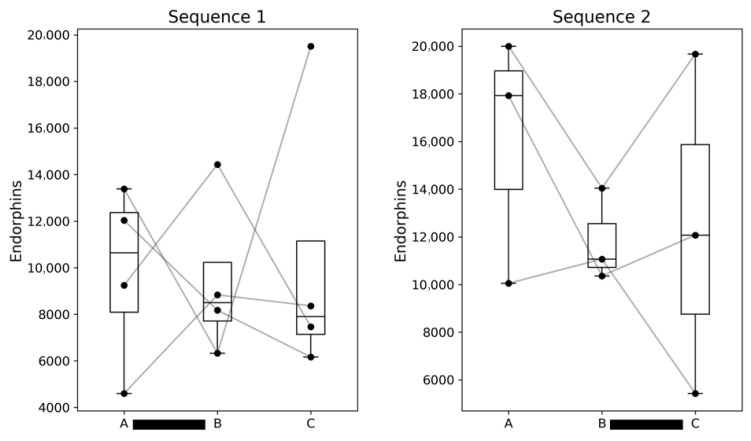
Salivary β-endorphin concentrations at each time point. Salivary β-endorphin concentrations (pg/mL) are presented as individual data points with boxplots at time points A, B, and C for Sequence 1 and Sequence 2. Black circles represent individual participants, and gray lines connect measurements from the same participant across time points. The boxes represent the interquartile range, the horizontal lines within the boxes indicate the median, and the whiskers extend to the minimum and maximum values. Bold horizontal lines indicate the music-listening periods (A–B in Sequence 1 and B–C in Sequence 2). No consistent pattern of change was observed across participants.

Salivary β-endorphin concentrations also showed inconsistent changes across participants, with no clear pattern associated with the intervention period (Figure 5). Because of the small sample size, findings were interpreted descriptively.

### 3.2. Subjective Experiences

Participants frequently associated their selected music with autobiographical memories, often recalling meaningful experiences from adolescence or earlier stages of life. Several participants also described increased motivation and positive psychological experiences. Some participants further reported greater engagement in daily activities, including work, driving, and volunteer activities. Overall, qualitative comments consistently referred to autobiographical memory recall, increased motivation, and greater engagement in daily life.

## 4. Discussion

This exploratory feasibility pilot study evaluated the feasibility of therapist-guided, self-directed personalized music listening in patients with fibromyalgia. The findings indicate that the intervention was feasible in daily life and that participants demonstrated substantial individual variability in their responses.

A central finding of this study is the substantial variability in individual responses. While some participants reported positive psychological experiences, others reported little perceived benefit or considered the intervention burdensome. This variability is consistent with previous reports indicating that the effects of music-based interventions are not uniform and may depend on individual preferences, emotional state, and contextual factors [7,10,14,15]. These findings support the view that music listening functions not as a standardized intervention, but as an individualized regulatory strategy that may facilitate emotional and behavioral adaptation in selected patients.

The absence of consistent reductions in pain scores is also consistent with the previous literature suggesting that music-induced analgesia is mediated by complex, context-dependent mechanisms [7,10]. Psychological processes such as attentional modulation and emotional regulation influence pain perception [11,12], and the gate control theory provides a framework for understanding how these factors may interact with nociceptive processing [13]. The absence of consistent pain reduction suggests that therapist-guided self-directed music listening should not be considered a uniformly effective analgesic intervention under the conditions examined in this pilot study.

In contrast, positive psychological experiences reported by some participants suggest that the primary effects of personalized music listening may be more strongly related to affective and motivational processes than direct analgesic mechanisms. This interpretation is consistent with neurobiological perspectives indicating that music engages reward-related and affective neural systems, including dopaminergic and opioidergic pathways [1,2,16,17]. However, the lack of consistent changes in salivary β-endorphin levels in this study suggests that such physiological effects may not be readily detectable using peripheral measures or may vary substantially across individuals. These findings do not support salivary β-endorphin as a consistent physiological marker of the psychological effects observed in this exploratory study.

Participants’ qualitative comments provide additional insight into these psychological effects. Several participants described memories associated with their selected music, including recollections from earlier periods of their lives. Others reported increased motivation and greater engagement in daily activities. These observations suggest that familiar favorite music may contribute to positive psychological experiences through autobiographical memory recall, which may facilitate emotional regulation, positive affect, and behavioral activation, rather than through direct analgesic effects alone, consistent with previous studies demonstrating that familiar music can evoke autobiographical memories and promote emotional regulation [4,5,18,19,20,21].

This study suggests that therapist-guided, self-directed, personalized music listening is feasible in daily life, including during periods when access to face-to-face healthcare is limited. Participants were able to incorporate music listening into their daily lives with guidance from a music therapist, highlighting the potential to integrate such interventions into routine care. At the same time, reports of burden and variability in adherence underscore the need for flexibility in intervention design, including consideration of frequency, duration, and individual readiness.

These findings are particularly relevant in the context of the COVID-19 pandemic, which has limited access to in-person healthcare and increased reliance on remote and self-managed interventions [22]. Therapist-guided but self-directed approaches, such as the one examined in this study, may offer a practical model for delivering supportive care under such constraints, while maintaining a degree of professional input.

Several limitations should be acknowledged. The small sample size limits the generalizability of the findings. In addition, the absence of a washout period introduces the possibility of carryover effects between study phases. Outcome measures relied partly on self-report, and salivary β-endorphin levels may not accurately reflect central opioid activity [17]. Variability in adherence and engagement was also not systematically quantified. Qualitative comments were based on the therapist field notes rather than on verbatim transcripts. Therefore, the findings should be interpreted as preliminary. Because of the exploratory nature of this pilot study, the results should not be interpreted as evidence of treatment efficacy but rather as preliminary observations intended to guide the design of future adequately controlled trials. Objective monitoring of adherence and a predefined qualitative analytical framework should also be incorporated into future studies.

Despite these limitations, this study contributes to the growing literature on personalized and self-directed interventions in integrative medicine. The results suggest that therapist-guided personalized music listening may be most appropriately conceptualized not as a uniform analgesic intervention, but as a feasible, individualized, context-dependent strategy that may facilitate participant-reported psychological experiences, autobiographical memory recall, motivation, and engagement in daily activities.

Further research should focus on identifying characteristics of responders and the patient populations most likely to benefit from therapist-guided personalized music listening. Larger studies are needed to evaluate clinical effectiveness further and clarify the role of personalized music listening within comprehensive pain management strategies. Future adequately controlled studies should incorporate larger sample sizes, objective monitoring of adherence, an appropriate washout period, and rigorous qualitative methods.

## 5. Conclusions

This exploratory feasibility pilot study suggests that therapist-guided, self-directed personalized music listening is feasible for patients with fibromyalgia in real-world daily life. Although no consistent effects on pain intensity or salivary β-endorphin levels were observed, some participants reported positive psychological experiences, including recall of autobiographical memories, increased motivation, and greater engagement in daily activities. These findings suggest that therapist-guided, self-directed personalized music listening may be a feasible supportive self-management approach rather than a uniformly effective analgesic intervention. Future adequately controlled studies with larger sample sizes, objective adherence monitoring, an appropriate washout period, and rigorous qualitative methodologies are warranted to further evaluate the feasibility, acceptability, and clinical effectiveness of this approach.

## Figures and Tables

**Table 1:** Participant characteristics and selected music categories.

Part 1										
ID	Age	Sequence	Pain		GHQ			JFIQ		
			AB	BC	A	B	C	A	B	C
1	57	ML-first	5	−	8	7	7	40	39	38
3	43	ML-first	4.84	4.19	6	0	0	56	32	28
5	46	ML-first	6.55	7.65	22	6	13	68	53	71
7	66	ML-first	2.97	2.17	9	8	8	61	72	56
4	75	non-ML-first	2.03	2.58	1	9	−	26	63	39
6	43	non-ML-first	−	−	1	−	−	−	40	49
8	69	non-ML-first	7.52	6.16	1	0	0	38	23	22
**Part** **2**										
**ID**	**Age**	**Endorphins (pg/mL)**					**Selected Music Category**			
		**A**	**B**		**C**					
1	57	4600.036	8832.337		8361.371		Classical/Film music			
3	43	13,387.575	6328.124		19,511.91		J-pop			
5	46	9246.257	14,433.644		7458.585		J-pop			
7	66	12,030.18	8173.253		6166.285		Showa pop			
4	75	>20,000	14,045.81		19,675.561		J-pop			
6	43	17,926.134	10,361.408		12,074.704		−			
8	69	10,050.265	11,069.318		5430.292		Classical			

Sequence indicates the order of intervention (ML-first or non-ML-first). Selected music categories were summarized at the genre level (e.g., J-pop, Showa pop [Japanese popular music from the Showa era], classical, film music). Pain scores were recorded daily and summarized as mean values for each period. AB and BC represent mean pain scores for the A–B and B–C intervals, respectively. In the ML-first sequence, AB corresponds to the ML period and BC to the non-ML period, whereas in the non-ML-first sequence, AB corresponds to the non-ML period and BC to the ML period. “–” indicates missing data resulting from unavailable measurements or incomplete self-reports.

## Data Availability

The data presented in this study are available from the corresponding author upon reasonable request. The data are not publicly available due to privacy restriction.

## Abbreviations

The following abbreviation is used in this manuscript:

ML	Music listening
